# Combined Beneficial Effect of Genistein and Atorvastatin on Adipogenesis in 3T3-L1 Adipocytes

**DOI:** 10.3390/biom11071052

**Published:** 2021-07-18

**Authors:** Dahae Lee, Ji-Youn Kim, Hae-Won Kim, Jeong-Eun Yoo, Ki Sung Kang

**Affiliations:** 1 College of Korean Medicine, Gachon University, Seongnam 13120, Korea; pjsldh@gachon.ac.kr; 2 Department of Obstetrics and Gynecology, College of Korean Medicine, Daejeon University, Daejeon 35235, Korea; april_023@daum.net (J.-Y.K.); ididkhw@nate.com (H.-W.K.)

**Keywords:** genistein, atorvastatin, adipocytes, adipogenesis

## Abstract

Genistein (4,5,7-trihydroxyisoflavone) is abundant in various dietary vegetables, especially soybeans, and is known to have not only an estrogenic effect but also an antiadipogenic effect. Atorvastatin (dihydroxy monocarboxylic acid) is a statin used to prevent heart disease. Although genistein and atorvastatin have been reported to possess antiadipogenic effects, their combined effects are still unclear. The aim of the current study was to explore whether the combination of genistein and atorvastatin at low concentrations significantly suppresses adipogenesis in a murine preadipocyte cell line (3T3-L1) compared to treatment with genistein or atorvastatin alone. Our results showed that cotreatment with 50 µM genistein and 50 nM atorvastatin significantly suppressed preadipocyte differentiation, whereas when each compound was used alone, there was no inhibitory effect. Additionally, cotreatment with genistein and atorvastatin significantly downregulated adipogenic marker proteins, including mitogen-activated protein kinases (MAPKs), peroxisome proliferator-activated receptor γ (PPARγ), CCAAT/enhancer-binding protein alpha (C/EBPα), glucocorticoid receptor (GR), and CCAAT/enhancer-binding protein β (C/EBPβ). This is the first evidence of the combined antiadipogenic effects of genistein and atorvastatin. Although additional experiments are required, combinational treatment with genistein and atorvastatin may be an alternative treatment for menopause-associated lipid metabolic disorders and obesity.

## 1. Introduction

Menopause is a stage in which menstruation stops owing to the aging of the ovaries [1]. Decreased estrogen secretion from the ovaries affects lipid metabolism [2]. Estrogen deficiency may downregulate the genes involved in fatty acid metabolism or lipid catabolism, which induces menopause-associated lipid metabolic disorders [3]. After menopause, the energy expenditure controlled by estrogen receptor activation is imbalanced and overall adiposity is increased, resulting in obesity [4,5].

Genistein (4,5,7-trihydroxyisoflavone), belonging to the isoflavone family, is abundant in various dietary vegetables, especially soybeans and fava beans, which are known as dietary phytoestrogens [6]. Genistein has been reported to show several biological activities such as anticancer effect [7], neuroprotective effect [8], anti-inflammatory effect [9] antiosteoporosis effects [10], and estrogenic [11] effect. It was also found that genistein decreased body weight, serum triglyceride, and liver lipid accumulation in ovariectomized rats, suggesting that genistein can effectively prevent adiposity and lipid disorders caused by estrogen deficiency [12].

Atorvastatin (dihydroxy monocarboxylic acid) is a statin-based representative medicine used as a primary treatment drug for dyslipidemia. It reduces the production of cholesterol in hepatocytes, thereby decreasing the serum levels of LDL cholesterol and triglycerides and increasing HDL cholesterol [13]. In addition, the antiadipogenic effect of atorvastatin in 3T3-L1 cells has been reported [14]. However, compared to genistein, there are few studies on the antiadipogenic effects of atorvastatin. After menopause, atorvastatin is usually prescribed for a long period of time to maintain serum lipid levels within the normal range. As the drug dose increases, there is a higher risk of side effects occurring, such as muscle pain, cognitive disorders, and diabetes [15]. Therefore, finding adjuvant drugs that can be used in combination with atorvastatin for adipogenesis may be an effective treatment strategy for menopause-related dyslipidemia and obesity.

The aim of the present study was to investigate whether the combination of genistein and atorvastatin would produce more effective antiadipogenic activity than genistein or atorvastatin alone in 3T3-L1 cells. We also evaluated the combined effects of genistein and atorvastatin on the regulation of the expression of key proteins involved in adipogenic pathways.

## 2. Materials and Methods

### 2.1. Cell Line and Materials

The 3T3-L1 mouse preadipocyte cell line was purchased from the American Type Culture Collection (Manassas, VA, USA). Dulbecco’s modified Eagle’s medium (DMEM) was obtained from Cellgro (Manassas, VA, USA). Fetal bovine serum (FBS), penicillin/streptomycin (P/S) antibiotics, and bovine calf serum (BCS) were purchased from Gibco (Gaithersburg, MD, USA). The EZ-Cytox cell viability assay kit, a tetrazolium salt (WST-1)-based colorimetric assay kit, was purchased from Daeil Lab Service (Seoul, South Korea). Phosphate-buffered saline (PBS), 1-methyl-3-isobutylxanthine (IBMX), Oil Red O solution, isopropanol, dexamethasone, formaldehyde solution, insulin, genistein, and atorvastatin were purchased from Sigma-Aldrich (St. Louis, MO, USA). Primary antibodies, including phospho-ERK (P-ERK), ERK, phospho-JNK (P-JNK), JNK, phospho-P38 (P-P38), P38, PPAR-γ, C/EBP-α, C/EBP-β, GR, glyceraldehyde 3-phosphate dehydrogenase (GAPDH), and horseradish peroxidase (HRP)-labeled anti-rabbit secondary antibodies were purchased from Cell Signaling Technology (Danvers, MA, USA). The ECL Plus Western blotting detection reagents were purchased from GE Healthcare (Piscataway, NJ, USA).

### 2.2. Cell Culture and Adipogenic Differentiation

The murine preadipocyte cell line (3T3-L1, American Type Culture Collection, Manassas, VA, USA) was grown in DMEM containing 10% BCS and 1% P/S antibiotics. For adipocyte differentiation, the culture medium was replaced with DMEM containing 10% FBS, 1% P/S antibiotics, 0.5 mM IBMX, 1 µM dexamethasone, and 5 µg/mL insulin. After incubation for two days, the culture medium was replaced with DMEM containing 10% FBS, 1% P/S antibiotics, and 5 µg/mL insulin every two days. The cells were incubated in DMEM containing 10% FBS and 1% P/S antibiotics every two days until the end of the experiment on day 8, as previously described [16]. During the process of adipogenic differentiation, genistein (25, 50 and 100 μM) and atorvastatin (25, 50 and 100 nM) were included individually or in combination (50 μM of genistein and 50 nM atorvastatin) in the culture medium.

### 2.3. Measurement of Cell Viability

The 3T3-L1 preadipocytes were grown in DMEM containing 10% BCS and 1% P/S antibiotics for 24 h and then treated with genistein (25, 50 and 100 μM) and atorvastatin (25, 50 and 100 nM) individually or in combination (50 μM of genistein and 50 nM atorvastatin). After 24 h, cell viability was investigated using the EZ-Cytox cell viability assay kit following previously described methods [17].

### 2.4. Oil Red O Staining

Oil Red O staining was performed on day 8 of cell differentiation. Cells were fixed in 4% paraformaldehyde solution. After 1 h, the fixed cells were gently washed with PBS three times and stained with Oil Red O solution (0.5%) in isopropanol. After 1 h, the stained cells were washed with distilled water three times. Oil Red O solution-stained lipid droplets were imaged using a light microscope and extracted with 100% isopropanol. The optical density was recorded at 520 nm using a microplate reader (PowerWave XS; Bio-Tek Instruments, Winooski, VT, USA).

### 2.5. Western Blot Analysis

The Western blot analysis was performed on day 8 of cell differentiation. Equal amounts of protein lysate from the 3T3-L1 cells were resolved by 10% sodium dodecyl sulfate–polyacrylamide gel electrophoresis. The samples were then transferred to nitrocellulose membranes. The membranes were probed with primary antibodies (1:1000) at room temperature for 1 h and probed with the HRP-labeled anti-rabbit secondary antibody (1:2000) under the same conditions. The probed blots were detected using ECL Plus Western blotting detection reagents. The protein expression of p-ERK, ERK, P-JNK, JNK, P-P38, P38, PPAR-γ, C/EBP-α, C/EBP-β, GR, and GAPDH was analyzed using a chemiluminescence system (FUSION Solo, PEQLAB Biotechnologie GmbH, Erlangen, Germany), as previously described [18,19].

### 2.6. Statistical Analysis

Statistical significance was determined using a one-way analysis of variance (ANOVA) and multiple comparisons with Bonferroni correction. Statistical significance was set at *p* < 0.05. All analyses were performed using SPSS Statistics ver. 19.0 (SPSS Inc., Chicago, IL, USA).

## 3. Results

### 3.1. Inhibitory Effects of Genistein and Atorvastatin on Adipogenesis in 3T3-L1 Preadipocytes

**Figure 1:**
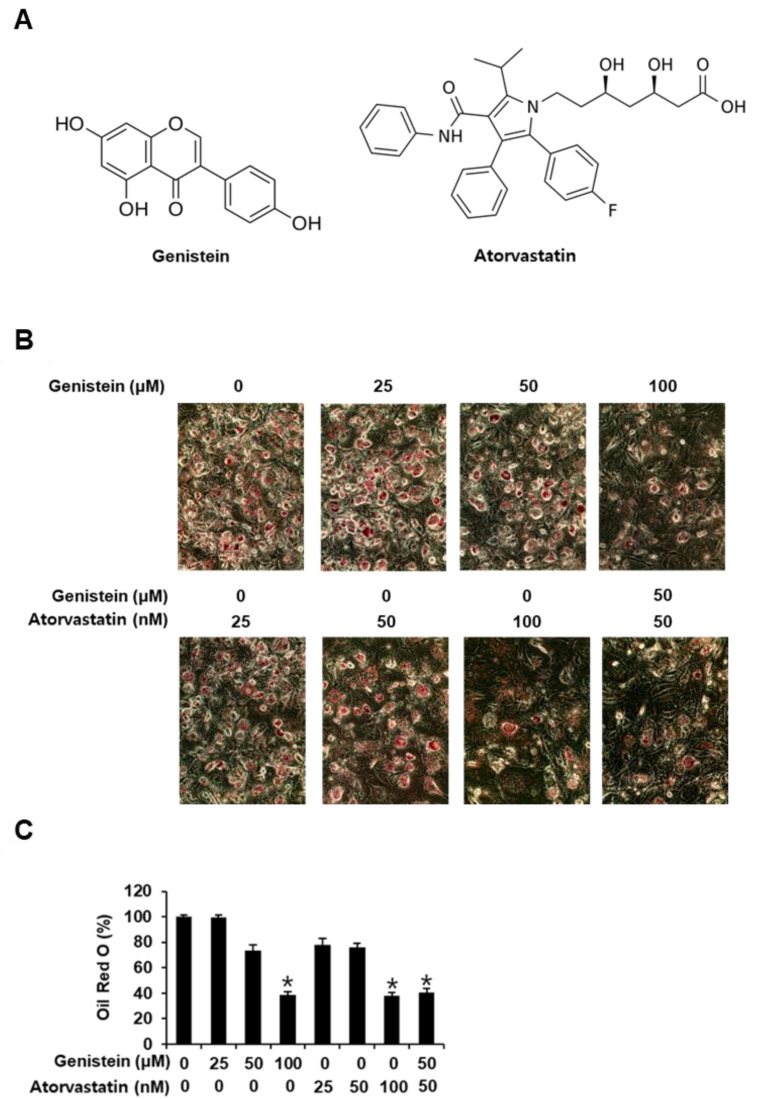
Inhibitory effects of genistein and atorvastatin on adipogenesis in 3T3L-1 preadipocytes. (**A**) Chemical structure of genistein and atorvastatin. (**B**) Images of the Oil Red O staining of differentiated 3T3L-1 cells photographed under an inverted microscope with 20X magnification on day 8 after treatment with genistein and/or atorvastatin. (**C**) Quantification of Oil Red O staining expressed as the percentage of the untreated control (n = 3 independent experiments, * *p* < 0.05, Kruskal–Wallis nonparametric test). Data are the mean ± SEM.

To determine the cytotoxicity of genistein, atorvastatin, and cotreatment with genistein and atorvastatin in 3T3-L1 preadipocytes, we used the EZ-Cytox cell viability assay kit. The cell viability assay showed that concentrations up to 100 µM genistein, 100 nM atorvastatin, and cotreatment (up to 100 µM genistein + 100 nM atorvastatin) did not affect the viability of 3T3-L1 preadipocytes after 24 h of incubation (data not shown). Their concentrations were used to determine their inhibitory effects on adipogenesis in 3T3-L1 preadipocytes. The differentiation of 3T3-L1 preadipocytes into mature adipocytes accompanied by intracellular lipid accumulation was evaluated using Oil Red O staining. As shown in Figure 1 C, treatment with 100 µM genistein alone, 100 nM atorvastatin alone, and cotreatment with 50 µM genistein and 50 nM atorvastatin significantly inhibited the differentiation of 3T3-L1 preadipocytes into mature adipocytes. Cells treated with 50 µM genistein alone showed a slight, but not significant, inhibition (26.62 ±  4.64% reduction) of the formation of red-labeled lipid droplets. Treatment with 50 nM atorvastatin alone showed a slight, but not significant, inhibitory effect (23.81 ±  2.81% reduction). However, cotreatment with 50 µM genistein and 50 nM atorvastatin resulted in a greater inhibition (59.47 ± 2.92% reduction) of the formation of red-labeled lipid droplets compared with either 50 µM genistein or 50 nM atorvastatin alone. Each treatment with 100 µM genistein and 100 nM atorvastatin, as well as the combined treatment of the 50 µM genistein and 50 nM atorvastatin, gave the same effect.

### 3.2. Effect of Genistein and Atorvastatin on the Expression of Proteins Involved in Adipogenesis in Differentiated 3T3L-1 Cells

**Figure 2:**
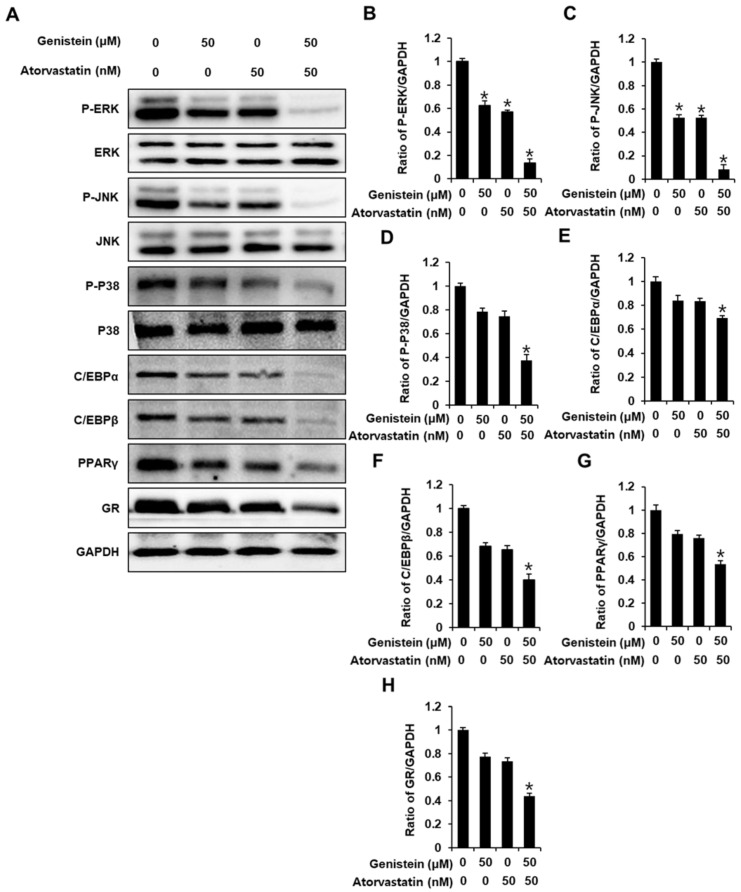
Inhibitory effects of genistein and atorvastatin on the expression of proteins involved in adipogenesis in differentiated 3T3L-1 cells. (**A**) The protein expression of phospho-extracellular signal-regulated kinase (P-ERK), ERK, phospho-c-Jun-N-terminal kinase (P-JNK), JNK, phospho-P38 (P-P38), P38, peroxisome proliferator-activated receptor γ (PPARγ), CCAAT/enhancer-binding protein alpha (C/EBPα), C/EBPβ, glucocorticoid receptor (GR), and glyceraldehyde 3-phosphate dehydrogenase (GAPDH) in differentiated 3T3L-1 cells on day 8 after treatment with genistein and/or atorvastatin. (**B**–**H**) Analysis of the ratios of the band intensities of P-ERK, P-JNK, P-P38, PPARγ, C/EBPα, C/EBPβ, and GR compared with differentiated 3T3L-1 cells without treatment (n = 3 independent experiments, * *p* < 0.05, Kruskal–Wallis nonparametric test). Data are the mean ± SEM.

To examine how genistein and atorvastatin inhibited adipogenesis in 3T3-L1 cells, we used a Western blot analysis to examine the expression of adipogenic marker proteins, including extracellular signal-regulated kinase (ERK), c-Jun-N-terminal kinase (JNK), P38, peroxisome proliferator-activated receptor γ (PPARγ), CCAAT/enhancer-binding protein alpha (C/EBPα), glucocorticoid receptor (GR), and CCAAT/enhancer-binding protein β (C/EBPβ). Treatment with either 50 µM genistein or 50 nM atorvastatin slightly inhibited the expression of adipogenic marker proteins in differentiated 3T3L-1 cells compared with the untreated controls. Cotreatment with genistein and atorvastatin suppressed the expression of these proteins even further (Figure 2A–H). This suggested that the cotreatment of genistein and atorvastatin was effective in downregulating adipogenic marker proteins during adipocyte differentiation for eight days.

## 4. Discussions

In the present study, we evaluated whether the combination of genistein and atorvastatin produced more effective antiadipogenic activity in 3T3-L1 cells than genistein or atorvastatin alone. We found that, when administered individually, 100 µM genistein and 100 nM atorvastatin significantly decreased the differentiation of 3T3-L1 preadipocytes, whereas 50 μM genistein and 50 nM atorvastatin had little effect. These results are consistent with those of the previous studies. It was previously suggested that atorvastatin exhibited a maximal inhibitory effect on the differentiation of 3T3-L1 preadipocytes at 100 nM [20]. In addition, the differentiation of 3T3-L1 preadipocytes was almost inhibited by 100 μM genistein [21,22]. However, no study has hitherto examined the combined effects of genistein and atorvastatin on the differentiation of 3T3-L1 preadipocytes. We found that cotreatment with 50 µM genistein and 50 nM atorvastatin caused greater inhibition of the differentiation of 3T3-L1 preadipocytes than 50 µM genistein or 50 nM atorvastatin alone. This indicated that cotreatment with genistein reduced the effective concentration of atorvastatin on the inhibition of adipogenesis. Thus, its combined effects may lead to reduced side effects, such as muscle pain or liver damage, after the long-term administration of atorvastatin to patients [23].

To further explore these effects, we evaluated the combined effects of genistein and atorvastatin on the regulation of the expression of key proteins involved in adipogenic pathways. The combination of 50 µM genistein and 50 nM atorvastatin, compared to 50 µM genistein or 50 nM atorvastatin alone, produced stronger inhibition of the adipogenic marker proteins, including the MAPKs (ERK, JNK, and P38), PPARγ, C/EBPα, GR, and C/EBPβ. Our results are in agreement with those of previous studies, where treatment with either 100 µM genistein or 100 nM atorvastatin alone inhibited adipogenesis via the inhibition of C/EBPβ and PPARγ in 3T3L-1 cells [20,21,24]. However, the detailed antiadipogenic mechanisms of genistein and atorvastatin and their combined effects are not yet completely clear. The MAPKs (ERK, JNK, P38), PPARγ, C/EBPα, GR, and C/EBPβ are linked to each other and play an important role in each stage of adipocyte differentiation [25].

In response to adipogenic signals, ERK, P38, and GR have been shown to promote the nuclear localization of C/EBPβ [26,27,28]. Glucocorticoids are steroid hormones that promote adipocyte differentiation via intracellular GR [29]. The transcription factor C/EBPβ is a key early regulator of adipogenesis [30]. It induces the activation of two master adipogenic transcription factors, PPAR-γ and C/EBPα [31]. Although the role of JNK in the stages of adipogenesis is uncertain, previous studies have shown that PPARγ transcriptional activity is modulated by JNK [32,33]. In the terminal differentiation of adipocytes, the transcriptional crosstalk between PPARγ and C/EBPα promotes the accumulation and storage of lipids in the adipocytes. In addition, they maintain a fully differentiated state [34,35,36].

The lack of estrogen after menopause induces lipid profile changes, which increases the risk of developing dyslipidemia, obesity, metabolic syndrome, and type 2 diabetes [31]. In a previous study, genistein exhibited excellent estrogen-like activity and estrogen receptor binding ability [32]. It has also been widely reported to have antiadipogenic effects [15,18,33,34,35]. Moreover, genistein treatment effectively modulated the plasma lipid indices in postmenopausal women with hyperlipidemia [36]. In this study, the combination of genistein and atorvastatin showed effects on the inhibition of adipogenesis in 3T3-L1 cells, and we assumed that genistein could be an adjuvant drug of atorvastatin for dyslipidemia and metabolic syndrome induced by a lack of estrogen.

**Figure 3:**
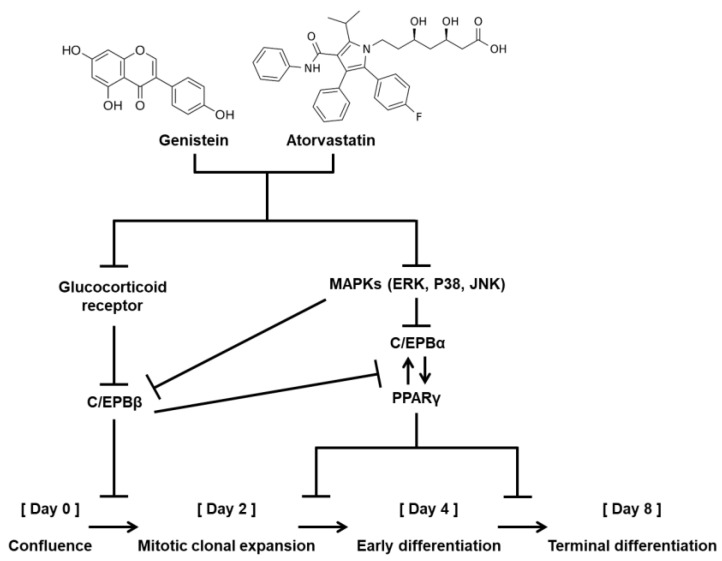
Schematic illustration of the underlying mechanism of the antiadipogenic effect of genistein and atorvastatin in 3T3-L1 preadipocytes.

Taken together, our data demonstrate that the antiadipogenic activity of genistein is synergized by cotreatment with atorvastatin. These antiadipogenic effects were associated with PPARγ and C/EBPα by regulating the MAPKs (ERK, JNK, and P38), GR, and C/EBPβ in 3T3-L1 adipocyte differentiation (Figure 3). Although further studies are needed, genistein and atorvastatin may be useful in the development of therapies for menopause-associated lipid metabolic disorders. However, lack of knowledge on natural product–drug interactions is a limitation of the current study that needs to be clarified in future animal experiments. Many cardiovascular drugs such as statins have been known to act as substrates and inhibitors of the solute carrier transporters and the ATP-binding cassette transporters. In addition, a growing number of studies on natural products (e.g., *Gingko biloba*, danshen) show that they are also substrates and inhibitors of drug transporters. Thus, assessment of natural product–drug interactions is important when considering clinical application [37,38].

## 5. Conclusions

Collectively, genistein and atorvastatin were previously reported to possess antiadipogenic effects. In addition, our results provide interesting information that the cotreatment of genistein and atorvastatin showed more potent inhibitory effects on adipogenesis than individual treatments, where cotreatment with genistein and atorvastatin, compared with genistein or atorvastatin alone, showed stronger inhibition of protein expression, including MAPKs (ERK, ERK, JNK, and P38), C/EBPα, C/EBPβ, PPARγ, and GR, which suggests greater antiadipogenic activity. Although further studies are needed to evaluate the antiadipogenic effects of genistein and atorvastatin on in vivo metabolism, this study provides a potentially useful therapeutic combination for menopausal patients at risk of lipid metabolic disorders and obesity.
